# Association of Hashimoto's thyroiditis with thyroid cancer

**DOI:** 10.1530/ERC-14-0258

**Published:** 2014-12

**Authors:** G Azizi, J M Keller, M Lewis, K Piper, D Puett, K M Rivenbark, C D Malchoff

**Affiliations:** 1 Wilmington Endocrinology, 1717 Shipyard Boulevard, Suite 220, Wilmington, North Carolina, 28403, USA; 1 Wilmington Pathology Associates, 1915 South 17th Street, Suite 100, Wilmington, North Carolina, 28401, USA; 2 Children's Hospital Colorado, 13123 East 16th Avenue, Aurora, Colorado, 80045, USA; 3 Carolina Arthritis, 1710 South 17th Street, Wilmington, North Carolina, 28401, USA; 4 University of Connecticut Health Center, 263 Farmington Avenue, Farmington, Connecticut, 06030, USA

**Keywords:** Hashimoto's thyroiditis, thyroid cancer, thyroid nodule, FNAB, TgAb

## Abstract

This prospective study investigates the relationship between Hashimoto's thyroiditis (HT) and thyroid cancer (TC) in patients with thyroid nodules (TNs). We prospectively examined 2100 patients with 2753 TNs between January 5, 2010 and August 15, 2013. A total of 2023 patients with 2669 TNs met the inclusion criteria of TN ≥5 mm and age ≥18 years. Each patient had blood drawn before fine-needle aspiration biopsy (FNAB) for the following measurements: TSH, free thyroxine, free tri-iodothyronine, thyroid peroxidase antibody (TPOAb), and antithyroglobulin antibody (TgAb). Diagnosis of TC was based on pathology analysis of thyroidectomy tissue. The associations of TC with the independent variables were determined by univariate and multivariate logistic regression analysis and reported as adjusted odds ratio (OR) with 95% CI. A total of 248 malignant nodules were found in 233 patients. There was an association of TC with both increased serum TgAb concentration and age<45 years. An elevated serum TgAb concentration was found in 10.2% of patients (182 of 1790) with benign nodules as compared with 20.6% of patients (48 of 233) with malignant nodules (*P*≤0.0001). TgAb (OR=2.24: CI=1.57, 3.19) and TSH ≥1 μIU/ml (OR (95% CI)) OR: 1.49 (1.09, 2.03) were significant predictors of TC in multivariate analysis controlling for age and gender. TC was not associated with serum concentrations of TPOAb. In patients with TN, elevated serum concentration of TgAb and TSH ≥1 μIU/ml are independent predictors for TC. The association between HT and TC is antibody specific.

## Introduction

Hashimoto's thyroiditis (HT) is the most common autoimmune thyroid disease and the most common cause of hypothyroidism (Jankovic *et al*. 2013). Epidemiological and histological data indicate that thyroid cancer (TC) frequently occurs in the context of one of the most common autoimmune thyroid diseases, HT, and that TC is frequently infiltrated by inflammatory-immune cells (Guarino *et al*. 2010).

HT is characterized by infiltration of the thyroid gland by inflammatory cells. This often leads to hypothyroidism due to destruction and eventual fibrous replacement of the parenchymal tissue. The relationship between HT and papillary carcinoma (PC) was first proposed by Dailey *et al*. (1955). Since this initial description, the association between the diseases has been repeatedly reported and highly debated in the literature and remains controversial (Cunha *et al*. 2011).

A relationship between chronic inflammation and cancer was first proposed by Virchow (1863) and has been sustained by clinical and epidemiological evidence. The most compelling evidence is the association between: i) intestinal chronic inflammatory diseases (Crohn's disease and ulcerative rectocolitis) and adenocarcinoma of the colon; ii) chronic HBV or HCV hepatitis and liver carcinoma; iii) *Heliobacter pylori*-induced chronic gastritis and gastric carcinoma; iv) asbestosis and mesothelioma; v) chronic obstructive pulmonary disease and lung cancer; and vi) scleroderma and carcinoma of the breast and lung (Guarino *et al*. 2010, Joseph *et al*. 2014).

Two recent retrospective studies have reported a positive association between HT and TC in patients presenting with thyroid nodules (TNs). In these two studies, antithyroglobulin antibody (TgAb), but not thyroid peroxidase antibody (TPOAb), was an independent risk factor for TC (Kim *et al*. 2010, Azizi & Malchoff 2011).

Based on these previous retrospective studies, we hypothesized that the association of HT with TC is antibody specific. Since a retrospective study's design may be subject to ascertainment bias and assay heterogeneity, we chose to further investigate the relationship between HT and TC by prospectively examining this relationship in TN patients using a multivariate approach to account for the possible influence of other variables including the levels of thyroid-stimulating hormone (TSH).

## Patients and methods

### Patients

The inclusion criteria were the presence of a single or multiple TNs ≥5 mm and age ≥18 years.

We prospectively evaluated 2100 patients with 2753 TNs from January 5, 2010 to August 15, 2013. All TNs were evaluated with a high-resolution ultrasound (US), fine-needle aspiration biopsy (FNAB), and, when indicated, by tissue pathology. All patients were evaluated by a single endocrinologist (G A) with more than 15 years of experience in thyroid US and ultrasound-guided FNAB.

The study protocol was approved by the Institutional Review Board of the New Hanover Regional Medical Center, Wilmington, NC. All patients gave written informed consent.

### Biochemical assays

At the time of initial US examination, the following factors were ascertained in all study subjects: gender, age, and number of US-determined TN. Treatment with any TSH-altering medication was documented. All patients had their blood drawn before FNAB for the following measurements: TSH, free thyroxine, free tri-iodothyronine, TPOAb, and TgAb. All measurements were performed by LabCorp (LabCorp Research Triangle Park-Center for Molecular Biology and Pathology, Esoteric Division, Research Triangle Park, NC, USA). Both TPOAb and TgAb levels were measured using Immulite 2000 technology by Siemens Medical Solutions Diagnostics (Brewster, NY, USA) utilizing purified antigen (TPO) immobilized to insoluble beads in a solid phase, enzyme-labeled chemiluminescent sequential immunometric assay. The reference range for TPOAb and TgAb levels was 0–34 and 0–40 IU/ml respectively. The analytical sensitivity for TPOAb and TgAb levels was 5 and 2.2 IU/ml respectively. Serum TSH level was measured at LabCorp by an electrochemiluminescence immunoassay developed by Roche Diagnostic. The reference range for TSH was 0.45–4.5 μIU/ml. Hypothyroidism is defined as TSH >4.5 μIU/ml without thyroid medication and in patients with a known history of hypothyroidism on thyroid hormone replacement. Hyperthyroidism is defined as TSH<0.45 μIU/ml without medication and in patients with prior history of hyperthyroidism on antithyroid medication.

### FNAB procedure

Before the FNAB, we obtained written informed consent for the procedure. The FNAB was performed under sterile conditions with US guidance to confirm accurate needle placement. Three passes were made of each lesion using 27-gauge needles. If there was no sample on visual inspection, we then used a larger needle (25-gauge). Onsite adequacy was not performed in this study. The samples were submitted for cytologic evaluation in accordance with the Bethesda system for reporting thyroid cytopathology, resulting in diagnostic Bethesda categories (BC) I (nondiagnostic or unsatisfactory), II (benign), III (atypia of undetermined significance or follicular lesion of undetermined significance), IV (follicular neoplasm or suspicious for follicular neoplasm), V (suspicious for malignancy), or VI (malignant) (Cibas & Ali 2009). For patients with BC I cytology, a second FNAB was offered.

### Selection for thyroidectomy

Surgical resection was recommended for patients with FNAB results that were positive for malignancy, highly suspicious for PC or follicular neoplasm, or follicular cells of undetermined significance. In addition, thyroid surgery was also recommended for patients with two non-diagnostic or benign FNABs with two or more US features suggestive of TC (irregular margins, microcalcifications, hypoechoic pattern) or increased size.

### Statistical analysis

The statistical analysis was performed by Dr M N Kuchibhatla (Associate Professor of Biostatistics and Director for Center for Aging, Duke University Department of Statistics) using SPSS/SAS program (SAS Institute, 2009. Statistical applications software: Release 9.2. SAS Institute, Inc., Cary, NC, USA). Means and s.d. were used to summarize quantitative variables, while frequencies and percentages were used to summarize qualitative data. Bivariate associations with TC were examined using non-parametric Wilcoxon tests for quantitative variables and *χ* ^2^ tests for categorical variables. Univariate association of independent variables (age, gender, elevated TgAb, elevated TPO, euthyroid normal antibodies, hyperthyroid, hypothyroid, multinodular goiter (MNG) gland, single nodule gland, thyroid medication, history of head/neck radiation, and family history of TC) on diagnosis of cancer was determined using logistic regression. Multivariable logistic regressions adjusting for age and gender were used to examine associations between TC and each of the variables and serum concentrations of TSH, TgAb, and TPOAb. A probability of <0.05 was considered statistically significant.

## Results

### Patients and TNs

**Figure 1:**
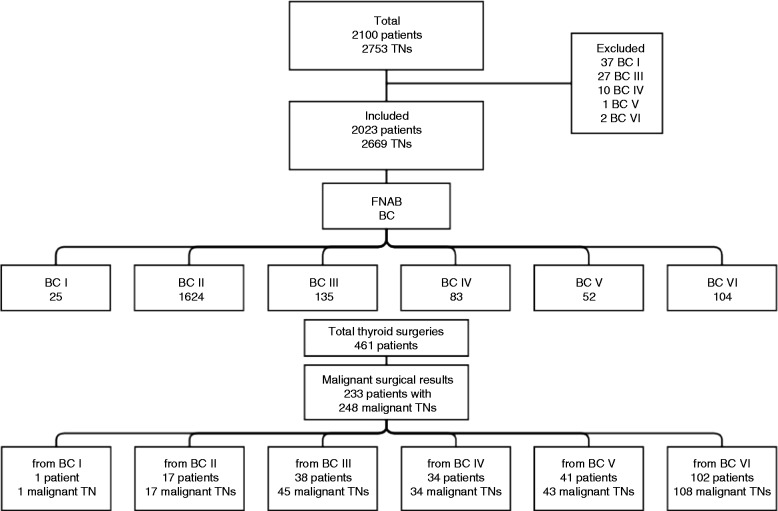
Flow chart for FNAB and surgical outcome.

Figure 1 summarizes the 2100 patients with 2753 TNs who were evaluated between January 5, 2010 and August 15, 2013. There were 77 patients with 84 TNs who were not included in the statistical analysis for one or more of the following reasons: patient moved away, patient refused recommended surgery, or patient did not return for follow-up. Of the 77 patients, 37 patients were excluded BC I, 27 were BC III, ten were BC IV, one was BC V, and two were BC VI.

The remaining 2023 patients with 2669 TNs were included in the statistical analysis. There were 1150 TNs that were <10 mm in greatest diameter, and the remaining 1519 TNs were ≥10 mm. There were 1348 patients who had FNAB of a single TN and 675 patients who had FNAB of two or more TNs.

### FNAB cytology

Figure 1 summarizes the FNAB cytopathology results of the 2023 patients. For patients with more than one nodule, the highest grade of BC was reported. 1624 patients were BC II, 135 patients were BC III, 83 patients were BC IV, 52 patients were BC V, and 104 patients were BC VI. Among patients with BC I, 25 remained in the study because they had surgery.

### Surgical pathology

Thyroidectomy was performed on 461 patients. Of these, 374 had abnormal FNAB (BC III, IV, V, or VI) and 25 had non-diagnostic FNAB (BC I). 62 patients with benign FNAB (BC II) had surgery due to worrisome US features or a size large enough to warrant surgery. Of the 461 patients who had surgery, 233 patients with 248 malignant TNs were diagnosed with TC. Figure 1 summarizes the number of patients assigned to each FNAB BC category.

TC was histologically confirmed in 233 patients: 45 patients had single nodule gland and 188 patients had MNG. Among those patients with MNG, 121 (64%) TCs were in dominant TN and 67 (36%) of TC were in non-dominant TN.

Of the 248 malignant TNs, 149 were PC, 83 were follicular variant of PC, four were medullary carcinoma, and 12 were follicular carcinoma. From the 248 malignant TNs, 104 were <10 mm diameter in size and 144 were ≥10 mm diameter in size. There were 44 follicular adenomas on surgical pathology in this study.

### Univariate associations with TC and surgery

As expected, young age was a risk factor for TC (Tables 1 and 2 respectively). The mean age of patients with benign nodules was 49.05 years (15.93%) and was greater than that for patients with malignant nodules, which was 45.33 years (15.29%) (*P*=0.0007). The malignancy rate among males was higher than females, but did not reach statistical significance most likely due to high number of female patients (84%) in this study.

There is an association between TC and TgAb. Serum TgAb concentration was elevated above the normal range in 20.6% (*n*=48) of the TC patients as compared with 10.17% (*n*=182) of the patients with benign nodule (*P*<0.0001). In contrast, TC was not associated with TPOAb concentration. Serum TPOAb concentration was elevated in 31.33% (*n*=73) of the TC patients and 26.7% (*n*=478) of the benign nodule patients (*P*=0.1356).

There was a trend for TC to be associated with family history of TC and with head/neck radiation, but these did not reach statistical significance.

Table 2 demonstrates that there was no significant association of surgical intervention with any of the biochemical variables, indicating that knowledge of serum antibody and TSH concentrations did not influence the investigator's decision to resect a nodule. Therefore, no selection bias for surgical resection influenced the final association of TC with TgAb.

### Multivariate associations with TC

In the multivariate analysis, controlling for age and gender, TgAb (OR (95% CI)): OR: 2.24 (1.57, 3.19) was a significant predictor of cancer while TPOAb (OR (95% CI)): OR: 1.19 (0.88, 1.61) was not (Table 3). In the initial analysis for this study, when divided into four prespecified groups, TSH was not found to be a predictor of TC. In the multivariate analysis, none of the TSH groups were a risk factor for TC. TSH groups included: 0 to 0.45 μIU/ml; >0.45 to ≤2 μIU/ml; >2 to ≤4.5 μIU/ml; and the final TSH group was patients with TSH >4.5 μIU/ml. However, when we retrospectively repeated the analysis with a single cutoff, TSH >1 μIU/ml was a predictor for higher risk for cancer (OR (95% CI)) OR: 1.49 (1.09, 2.03). In an age and gender-adjusted multivariate logistic regression analysis of subjects who were not taking any TSH-altering medication, undetectable TgAb level remained a significant predictor of cancer (OR (95% CI)): OR: 2.28 (1.42, 3.66).

Table 4 shows a detailed breakdown of all patients included in the study. We divided patients with normal TgAb level by those with undetectable TgAb level (Ref: 0) and those with detectable TgAb level, but still within normal range (>0 to <40). We had 1706 patients with undetectable TgAb level; among those 175 patients had TC and 1531 patients had no cancer. We had 89 patients with TgAb level with detectable but, still within normal range; among those 15 had TC and 74 had no cancer. We then grouped patients with elevated TgAb levels by those with TgAb >40 to <200 and patients with TgAb ≥200. In the first group, we had a total of 122 patients with 24 having cancer and 98 without TC. In the final group with TgAb ≥200, we had 106 patients. Among those patients, 19 had TC, and 87 had no cancer. Table 5 gives a detailed breakdown of the number of patients by TSH concentration.

## Discussion

Our prospective study of patients with TN demonstrates that an elevated serum TgAb concentration is an independent predictor for TC. Serum TPOAb concentration was not a predictor of TC in either univariate or multivariate analyses.

This prospective study was undertaken to further investigate the risk of thyroid inflammation and TC. It confirms two retrospective studies that suggested this association (2, 22). For the study's assay, the normal reference range for TgAb concentration was 0 to 40 IU/ml. In the multivariate analysis, we found that patients with detectable but normal TgAb concentrations (>0 to <40 IU/ml) had an (OR (95% CI)): OR: 1.70 (0.95, 3.03). This finding suggests that lowering the cutoff value for TgAb or TPOAb may reduce the false negative misclassifications, as noted by Spencer (2011) and Spencer *et al*. (2011). This statistically non-significant trend for association with TC would be of interest to investigate in a larger study with greater statistical power to determine if the risk of TC with increasing TgAb is present at any TgAb concentration or if there is a threshold TgAb concentration below which there is no increased risk of TC.

There were 114 patients with TC who had normal thyroid function and normal thyroid antibodies (TPOAb <34 IU/ml and TgAb <40 IU/ml). In this group, 17 patients (15%) had histopathologic changes in HT. This finding may indicate that the prevalence of HT in TC might be higher than expected.

A recent review by Jankovic *et al*. (2013) has revealed significant differences in the prevalence and the risk ratio of TC in HT specimens obtained via FNAB vs thyroidectomy. All eight FNAB studies (Crile & Hazard 1962, Holm *et al*. 1985, Carson *et al*. 1996, Erdogan *et al*. 2009, Matesa-Anić *et al*. 2009, Anil *et al*. 2010, Mukasa *et al*. 2011) with negative association between HT and TC have a low prevalence of TC, 0–2.9%, but the prevalence of surgical thyroidectomy studies ranges from 9.4 to 36% (Ott *et al*. 1985, Singh *et al*. 1999, Cipolla *et al*. 2005, Kurukahvecioglu *et al*. 2007, Larson *et al*. 2007, Repplinger *et al*. 2008, Bradly *et al*. 2009, Mazokopakis *et al*. 2010, Siriweera & Ratnatung 2010, Jankovic *et al*. 2013).

 The prevalence of TC in this prospective study was 11.5% for patients and 9.2% for TNs, which is within the 5–15% TC risk expected for TNs (Cooper *et al*. 2009). Approximately 41% of the TCs were <10 mm in diameter, which is not unexpected for a US study that relies heavily on US for nodule detection. The risk of TC for each FNAB BC group was similar to the expected risk (Cooper *et al*. 2009). Since the population under investigation is similar to those reported in the literature, it seems unlikely that a selection bias, error in FNAB classification, or unique risk factor is influencing the reported associations.

The effect of TSH, family history, and external radiation were examined. We did observe a statistically significant effect of TSH on TC risk in TN patients when we retrospectively analyzed our results using a serum TSH cutoff (TSH >1 μIU/ml) that had not been prespecified. As reviewed by McLeod *et al*. (2012), the association of TSH with TC is weak when the effect of autoimmune thyroid disease is incorporated into the multivariate analysis (Gul *et al*. 2010, Jin *et al*. 2010). When divided into four groups, TSH was not a predictor for TC. Where TSH is divided into four groups (with <0.45 μIU/ml as the reference group) the lack of effect may be due to inadequate power. This result was seen in two previously published studies (Kim *et al*. 2010, Azizi & Malchoff 2011). Similarly, the lack of an effect of family history and radiation exposure on the risk of TC is due to the small number of patients with these risk factors, limiting the power of this study. The association of young age and trend for association with male gender are known associations that are reproduced in this study.

The association of TgAb with malignancy in patients with nodule has been found in some studies (Kim *et al*. 2010, Azizi & Malchoff 2011), but not all (Boelaert 2009). Boelaert evaluated TPOAb, but not TgAb (Boelaert *et al*. 2006). Haymart *et al*. (2008, 2009) reported that independent of age, higher TSH level was associated with increased incidence of TC and extrathyroidal extension of disease. In their study, neither TPOAb nor TgAb status was reported. The failure of some studies to identify this relationship may be related to the variability of the TgAb assay. TgAb assays are highly variable as a consequence of many factors, including the heterogeneity of TgAb (Latrofa *et al*. 2012).

The relationship between inflammation and TC is complex and still not completely understood (Guarino *et al*. 2010). Both retrospective studies and now this prospective study of TN patients suggest that the association of HT with TC is antibody specific (Kim *et al*. 2010, Azizi & Malchoff 2011). The mechanism leading to this association is not known. TgAb may have a tumorigenic effect or be closely associated with a specific tumorigenic inflammatory response. Alternatively, the thyroglobulin molecule in TC may be more antigenic due to altered processing or an acquired or germline mutation in the primary structure. It is of interest that the TPOAb is not associated with a risk of TC. TPOAb is believed to fix complement and is a more sensitive marker for HT and hypothyroidism than TgAb. Perhaps the cytotoxic effect of TPOAb is that it protects against the tumorigenic effect of thyroid inflammation. It is also of interest that the development of malignancy in scleroderma is also antibody specific. Further investigations into this phenomenon of antibody specificity may lead to a better understanding of tumorigenesis (Joseph *et al*. 2014).

The present study has several strengths. The biochemical measurements and patient phenotypic data were all collected prospectively to eliminate selection bias, which is confirmed by the lack of association of surgical intervention with biochemical analysis. All measurements of TSH, TgAb and TPOAb were made at the same laboratory and using the same methodology for each patient to eliminate any interference from variability of different assay methodologies. Finally, only 3.7% of the initial 2100 patients seen were excluded from the study due to previously discussed reasons. This small fraction is unlikely to affect the outcome of this study. Since this is a cross-sectional association study, it does not directly address the mechanism that explains the association of TgAb and TC.

In summary, elevated serum TgAb level was associated with an increased risk of TC in patients with TN. In single cutoff analysis, TSH ≥1 μIU/ml is a significant predictor of TC. Serum TPO was not associated with TC. We conclude that the association of HT with TC is antibody specific.

## Figures and Tables

**Table 1:** Univariate analysis: related variables with thyroid cancer for all patients enrolled in the study

**Variable**	**No cancer *n* (%)/mean** (s.d.) (*N*=1790)	**Cancer *n* (%)/mean** (s.d.) (*N*=233)	***P* value**
Age (mean/s.d.)	49.05 (15.93%)	45.33 (15.29%)	0.0007
*N* (%)			
Female	1503 (83.97%)	190 (81.55%)	0.3467
Elevated TgAb	182 (10.17%)	48 (20.6%)	<0.0001
Elevated TPOAb	478 (26.7%)	73 (31.33%)	0.1356
Euthyroid normal antibodies	935 (52.23%)	114 (48.93%)	0.3419
Hyperthyroid	189 (10.56%)	18 (7.73%)	0.1795
Hypothyroid	516 (28.83%)	72 (30.9%)	0.5118
MNG gland	1502 (83.91%)	188 (80.69%)	0.2119
Single nodule gland	288 (16.09%)	45 (19.31%)	0.2119
Thyroid medication	508 (28.43%)	69 (29.61%)	0.7062
History of head/neck radiation	24 (1.37%)	6 (2.59%)	0.1556
Family history of TC	90 (5.03%)	18 (7.73%)	0.2263

**Table 2:** Univariate analysis: related variables with thyroid surgery for all patients enrolled in the study

**Variable**	**No surgery *n* (%)/mean** (s.d.) (*N*=1582)	**Surgery *n* (%)/mean (s.d.)** (*N*=461)	***P* value**
Age (mean/s.d.)	49.41 (16.04)	45.93 (15.11)	<0.0001
*N* (%)	1582 (1582)	461 (461)	
Female	1318 (84.38)	375 (81.34)	0.1213
Elevated TgAb	167 (10.69)	63 (13.67)	0.0771
Elevated TPOAb	433 (27.72)	118 (25.6)	0.3680
TSH concentration	2.44 (8.28)	1.92 (2.09)	0.9482
Euthyroid normal antibodies	804 (51.47)	245 (53.15)	0.5276
Hyperthyroid	151 (9.67)	56 (12.15)	0.1226
Hypothyroid	468 (29.96)	120 (26.03)	0.1024
MNG gland	1305 (83.55)	385 (83.51)	0.9868
Single nodule gland	257 (16.45)	76 (16.49)	0.9868
Thyroid medication	455 (29.19)	122 (26.46)	0.2558
History of head/neck radiation	23 (1.47)	8 (1.74)	0.6864
Family history of TC	79 (5.06)	29 (6.29)	0.5838

**Table 3:** Age and gender-adjusted multivariate logistic regression predicting cancer

**Variable**	**Odds ratio**	**95% Lower CL**	**95% Upper CL**
Thyroglobulin antibody	2.24	1.57	3.19
TPO antibody	1.19	0.88	1.61
TgAb level (Ref: 0)			
>0 to <40	1.70	0.95	3.03
≥40 to <200	2.05	1.27	3.31
≥200	1.89	1.12	3.19
TSH level (Ref: <1)			
≥1	1.46	1.07	1.99
TSH level (Ref: 0 to 0.45)			
>0.45 to ≤2	1.16	0.74	1.81
>2 to ≤4.5	1.27	0.78	2.07
>4.5	1.23	0.65	2.35

All patients in the study with the following variables: TgAb, TPOAb and TSH level, once in a single cutoff analysis and once in multigroup analysis.

**Table 4:** Breakdown of patients by TgAb level in age and gender-adjusted multivariate logistic regression

**Cancer predicted by TgAb level**					
Frequency	Ref: 0	>0 to <40	≥40 to <200	≥200	Total
No cancer	1531	74	98	87	1790
Cancer	175	15	24	19	233
Total	1706	89	122	106	2023

A detailed breakdown of all patients included in the study. All patients were divided into two groups: those with undetectable TgAb (Ref: 0), and those with detectable but normal range TgAb (>0 to <40). The elevated antibody group was then further broken down into the following ranges: ≥40 to <200 and ≥200.

**Table 5:** Breakdown of patients by TSH level in age and gender-adjusted multivariate logistic regression

**Cancer predicted by TSH level**					
Frequency	Ref: 0 to 0.45	>0.45 to ≤2	>2 to ≤4.5	>4.5	Total
No cancer	232	995	438	125	1790
Cancer	26	128	61	18	233
Total	258	1123	499	143	2023

A detailed breakdown of all patients included in the study. All patients were divided based on TSH level.
